# Quantitative analysis of systemic perfusion and cerebral blood flow in the modeling of aging and orthostatic hypotension

**DOI:** 10.3389/fphys.2024.1353768

**Published:** 2024-08-01

**Authors:** Heming Cheng, Jifeng Dai, Gen Li, Dongfang Ding, Jianyun Li, Ke Zhang, Liuchuang Wei, Jie Hou

**Affiliations:** ^1^ Department of Mechanics, Kunming University of Science and Technology, Kunming, China; ^2^ Department of Hydraulic Engineering, Kunming University of Science and Technology, Kunming, China

**Keywords:** orthostatic hypotension, cerebral blood flow, systemic perfusion, dementia, artery aging

## Abstract

**Introduction:** Orthostatic hypotension (OH) is common among the older population. The mechanism hypothesized by OH as a risk factor for cognitive decline and dementia is repeated transient cerebral blood flow deficiency. However, to our knowledge, quantitative evaluation of cardiac output and cerebral blood flow due to acute blood pressure changes resulting from postural changes is rare.

**Methods:** We report a new fluid-structure interaction model to analyze the quantitative relationship of cerebral blood flow during OH episodes. A device was designed to simulate the aging of blood vessels.

**Results and Discussion:** The results showed that OH was associated with decreased transient cerebral blood flow. With the arterial aging, lesions, the reduction in cerebral blood flow is accelerated. These findings suggest that systolic blood pressure regulation is more strongly associated with cerebral blood flow than diastolic blood pressure, and that more severe OH carries a greater risk of dementia. The model containing multiple risk factors could apply to analyze and predict for individual patients. This study could explain the hypothesis that transient cerebral blood flow deficiency in recurrent OH is associated with cognitive decline and dementia.

## 1 Introduction

With the continuously aging population, cognitive decline and dementia are becoming increasingly important public health issues, and the incidence of Alzheimer’s disease (AD) is predicted to be triple by 2050 (Hebert et al., 2013). Similarly, the World Health Organization has predicted that the number of people diagnosed with dementia will increase from approximately 55 million to 139 million by 2050 (WHO, 2021). Dementia will bring serious economic and social burdens to humanity in the coming decades (Michalowsky et al., 2019).

The development of cognitive dysfunction and dementia is a complex, multifactorial process. Numerous studies have identified various risk factors for cognitive function (CF) decline and dementia, such as decreased cardiac output, heart failure, atrial fibrillation, anemia, OH, chronic hypotension, hypertension, resting heart rate, arterial vascular aging, and carotid atherosclerosis, among others (Cicconetti et al., 2004; Cremer et al., 2017; Hughes et al., 2020). OH has been suggested as one of these risk factors. This study aimed to assess the changes in cerebral blood flow (CBF) during OH and confirm the hypothesis regarding the mechanism of CBF reduction when OH occurs.

OH is a common disorder in the older population, with a prevalence rate ranging from 5% to 30% (Tilvis et al., 1996; Frewen et al., 2014), and more than 50% in institutionalized older populations (Vloet et al., 2005). In addition, OH is associated with an increased risk of cardiovascular morbidity (Angelousi et al., 2014; Milazzo et al., 2015; Juraschek et al., 2018) and all-cause mortality (Maule et al., 2012; Angelousi et al., 2014). The OH mentioned in this paper includes both neurogenic and non-neurogenic induced, as well as postprandial hypotension. Recent evidence suggests a bidirectional correlation between OH, cognitive impairment (CI), and dementia (Yap et al., 2008; Isik et al., 2019; Kleipool et al., 2019; Robertson et al., 2019; Soysal et al., 2019). Over the last few decades, several epidemiological studies have investigated the potential link between OH and CI (Min et al., 2018). However, the published findings are inconsistent due to the environment, genetics, and health status of individuals, so no substantive conclusions can be drawn (Min et al., 2018).

The most frequently reported mechanism is the reduced transient CBF hypothesis during OH episodes. Previous electroencephalography studies show CBF reduced in patients with OH (Elmstáhl and Rosén, 1997), and reduced CBF at resting blood pressure (BP) was confirmed using single photon emission computed tomography (Hayashida et al., 1996; Töyry et al., 1997). OH is usually considered harmful only when the compensatory mechanisms are inadequate. When cerebral autoregulation is impaired, it is less efficient in compensating for the decrease in cerebral perfusion pressure and fails to maintain adequate CBF, which may lead to ischemic brain damage (Liu and Zhang, 2012). CBF depends on systemic perfusion (SP). Unfortunately, the quantitative relationship between OH, SP, and CBF perfusion status has not been well studied. In addition, whether a subtle decrease in OH and cerebral perfusion directly affects CBF in humans is still not fully understood. Due to the influence of heredity, environment, etiology, disease severity, comorbidity and early intervention, there is heterogeneity in SP and CBF among individuals (Claassen et al., 2021), a model containing multiple risk factors is required to analyze and predict for individual patients. Therefore, the development of a model that can contain multiple risk factors for dementia and assess the SP and CBF is a topic worthy of study.

It has been shown that one of the main manifestations of age-induced arterial biomechanics changes is a significant decrease in arterial axial pre-stretch ratio (AAPSR) and aortic compliance (Horny et al., 2011; Coccarelli et al., 2018). In recent years, the effect of aging-induced AAPSR reduction on the mechanical properties of blood vessels has attracted much attention (Horny et al., 2014a; Horny et al., 2014b; Kamenskiy et al., 2016). These results have undoubtedly contributed to further understanding of the mechanical properties of elastic arteries during human aging. However, their studies focused on the effect of reduced AAPSR on vascular constitutive relationships and did not directly characterize the effect of reduced AAPSR on chronic disease in the elderly.

The main objectives of this study were as follows: first, from the biomechanical point of view, to establish a fluid-structure interaction model between multiple physiological parameters of human circulation and mean blood flow, to analyze the relationship between SP and CBF in the occurrence of OH. Second, the effects of arterial vascular aging on SP were interpreted using a stretch-inflation test of the porcine thoracic artery and human anatomical data. SP was calculated under different physiological parameters. Finally, the interaction between OH and other risk factors such as arterial aging, CVD, and carotid plaque on SP and CBF were analyzed.

## 2 Materials and methods

### 2.1 A new fluid-structure model of blood circulation with vascular aging

#### 2.1.1 Strain energy density of elastic arteries

Similar to the potential energy that drives water to flow downward, deformed elastic arteries store elastic potential energy. For clinical application, we describe the strain energy density increment (SEDI) with physiological variables that can be measured in clinical practice, such as the outer diameter and wall thickness of the aorta, systolic and diastolic blood pressure, blood viscosity, density and make the following assumptions. Previous studies have shown that the mechanical properties of the aorta can be approximated by linear elasticity within the physiological range (Cheng et al., 2022). Based on the circumferential stress-strain relationship, the SEDI (*∆u*) of the elastic blood vessels during the systolic and diastolic periods can be approximated as:

$$∆u=\varphi^{2}{\left(\alpha_{s}p_{s}-{\alpha_{d}p}_{d}\right)}^{2}/8E$$ (1)

where ∆u is the SEDI of the aorta, φ is the constraint coefficient of the aorta, $\alpha_{s\;}$ and $\alpha_{d}$ are the coefficients, $\alpha_{s}=D_{s}/h_{s}$ and $\alpha_{d}=D_{d}/h_{d}$ , E is the circumferential modulus of elasticity, and $D_{s}$ , $D_{d}$ , $h_{s}$ and $h_{d}$ are the systolic and diastolic inner diameters and wall thicknesses of the aorta, respectively. $p_{s},$ and ${\;p}_{d}$ are the systolic and diastolic blood pressure (SBP, DBP).

#### 2.1.2 A new fluid-structure interaction model of blood circulation with vascular aging

**FIGURE 1:**
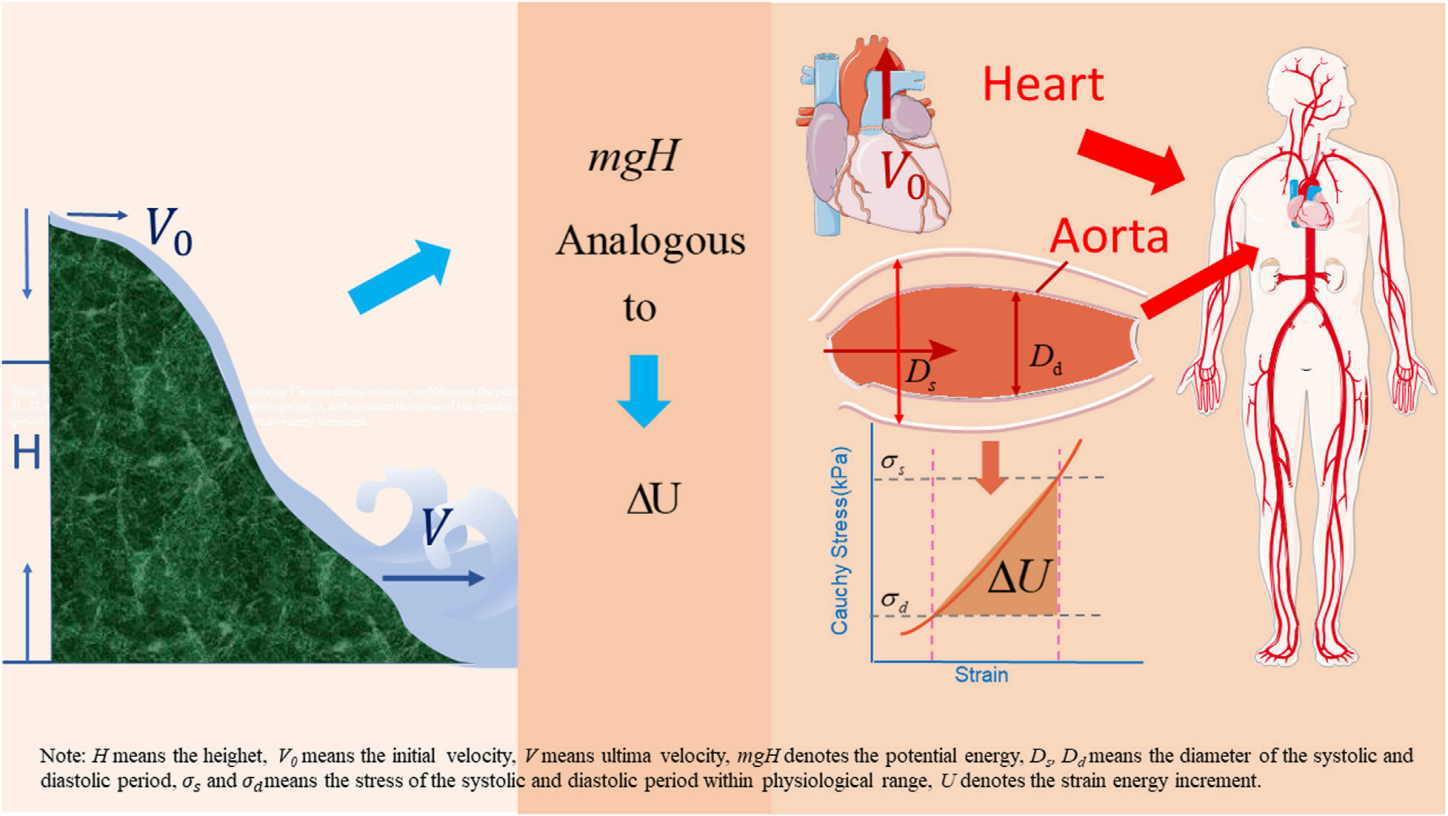
Analogous diagram of water flow and blood circulation.

Cheng et al. proposed a new understanding of human blood circulation and built a corresponding fluid-structure interaction model (Cheng et al., 2022). Unlike the Windkessl model, this model emphasizes that the heart pumps blood at an initial velocity to provide the initial kinetic energy for circulation. The strain energy then drives the blood flow. The work performed by the shear forces on the vessel walls is the energy dissipated by the blood flow. This new model is analogous to river flow. Rivers typically originate in high altitudes. The velocity of the water in the source area is extremely low, as is the corresponding output. Owing to the difference in the altitude of the locations, the river flows downward under the effect of potential energy (mgH). At high altitudes, many small streams converge to form larger rivers, and the flow velocity of the river depends on the initial kinetic energy of the source and altitude difference. In human circulation, cardiac output is analogous to the flow of water at the source of a river, and the strain energy (*∆u*) stored in the arteries is analogous to the potential energy of the river resulting from differences in altitude at different locations (see Figure 1). To reflect the effect of vascular aging, namely the decrease in the AAPSR with age, on blood supply, a fluid-structure interaction model should be established considering axial deformation.

The aortic blood flow is assumed to be laminar. In the human aorta, the amplitude of the Reynolds number Re is approximately 1,500, which is much lower than the critical value of 2,100 (Manning, 2012). To date, there is no experimental evidence of sustained turbulence in the human circulation (Thomas and Sumam, 2016). Throughout most of the cardiovascular cycle, the blood flow in the circulatory system can be characterized as laminar. In exceptional cases, when the heart contracts to its peak, some turbulence occurs in the blood flow in the aortic root and pulmonary trunk (Freis and Heath, 1964; Numata et al., 2016). Turbulent or highly disturbed blood flow occurs on the posterior side of diseased valves, at stenosis sites, or at the locations of implanted mechanical devices. The diastolic and systolic blood kinetic energy increments were calculated as follows:

$$\Delta K=\varphi \gamma \left(\alpha_{s}p_{s}-{\alpha_{d}p}_{d}\right)V^{2}/E$$ (2)

The dissipated energy increment of fluid flow $∆w_{\tau }$ (the increment of work done by blood and wall shear stress), is calculated as follows:

$$∆w_{\tau }=-\varphi \gamma \lambda \left(D_{s}-D_{d}\right)K^{2}V^{2}$$ (3)

λ is the friction loss factor of the elastic vessel (which is related to the flow state, fluid viscosity, and blood lipid levels), for laminar flow, calculated as λ=64/Re ( $\left.Re=VD/\mu \right)$ , in which *Re* is the Reynolds dimensionless coefficient, *μ* is the blood viscosity, and *K* is a coefficient. According to the Hamilton principle for fluid-structure interaction, from Eqs 1–3, the functional ( χ ) of the constructed aortic vessel per pulse unit length is represented as follows:

$$\chi =∭\varphi \gamma \left(\alpha_{s}p_{s}-{\alpha_{d}p}_{d}\right)V^{2}/EdV-∭{\varphi^{2}\left(\alpha_{s}p_{s}-{\alpha_{d}p}_{d}\right)}^{2}/8EdV$$

$$-\iint \left(D_{s}-D_{d}\right)\varphi \gamma \lambda K^{2}V^{2}dS$$ (4)

*V* is the flow distance per unit weight of blood per stroke through the arterial cross-section, defined as the characteristic velocity. According to the Hamilton’s principle for fluid-structure interaction, *V* can be obtained by the variational operation of *D*_*s*_ and *D*_*d*_. The following equation can be obtained:

$$V=\lambda_{z}\sqrt{\frac{\varphi h}{D\gamma \left(1+\beta \right)}\left(\alpha_{s}p_{s}-{\alpha_{d}p}_{d}\right)}$$ (5)

in which,

$$\beta =\frac{4E\lambda K^{2}\left(h_{d}-h_{s}\right)\;}{D\left(p_{s}-p_{d}\right)\varphi }$$ (6)

β is defined as the coupled energy dissipation coefficient, which is related to fluid viscosity, lipid levels, vessel geometry, pulse pressure, and artery stiffness. Thus, from Eqs 4–6, the mean blood flow *Q* per stroke through the cross-section can be calculated as follows:

$$Q=Q_{0}+∆Q=\lambda_{z}\left(1+\varphi \frac{DP}{Eh}\right)A_{f}\sqrt{\frac{\varphi h}{D\gamma \left(1+\beta \right)}\left(\alpha_{s}p_{s}-{\alpha_{d}p}_{d}\right)\;}$$ (7)

*A*_*f*_ is the initial blood flow area of the aorta. The $\lambda_{z}$ is defined as the ratio of the *in situ* length *l* to the *in vitro* length *l*_*0*_. *P* is the internal diameter of the aorta under mean pressure, $P=\left(2*p_{d}+p_{s}\right)/3$ , and h is the wall thickness under mean pressure. Combined with Eq. 7, the mean flow per minute ( $\bar{Q}$ ) through the arterial cross-section was calculated as follows:

$$\bar{Q}=n\lambda_{z}\left(1+\varphi \frac{DP}{Eh}\right)A_{f}\sqrt{\frac{\varphi h}{D\gamma \left(1+\beta \right)}\left(\alpha_{s}p_{s}-{\alpha_{d}p}_{d}\right)}$$ (8)

where *n* denotes the pulse frequency. The model expresses the coupled effects of multiple physiological variables on blood supply. It is also an approximate analytical solution for calculating the mean flow rather than a numerical solution.

### 2.2 Simulation tests of porcine vascular aging

Decrease in AAPSR is one of the manifestations of human arterial aging (vascular lesions excluded). From averages of normative data from 365 human beings’ autopsies, it is known that the AAPSR of human thoracic arteries is 1.33, 1.23, 1.08, 1.05, and 1.01 in 20, 30, 60, 70, and 80 year old populations, respectively (Horny et al., 2014a). The objectives of this simulation experiment were: 1) to confirm that the pre-stretched artery blood stores strain potential energy. 2) to characterize the changes in strain potential energy, elastic modulus and circumferential deformation of arterial vessels at different AAPSRs (simulating the *in vivo* decrease in AAPSR at different ages in humans). 3) to characterize the changes in strain potential energy, elastic modulus and circumferential deformation of arterial vessels at different AAPSRs.

#### 2.2.1 Materials

To improve human medical technology, animal models are key to evaluating mechanical, physiological, and pathological properties. Biomechanical studies have shown that the mechanical properties, geometric dimensions, and tissue composition of pigs are similar to those of humans, for example, the planar tensile test and stretch-inflation test results of human artery tissues share the same curve shape (Peña et al., 2019). The stress-strain method mentioned in this paper is universally applicable not only to porcine arterial vessels but also to human arterial vessels. The results of this study can be applied to human blood flow analyses. The AAPSR of arterial vessels decreases with the increase of age in humans, which is one of the manifestations of human artery aging (Horny et al., 2014b). Ten fresh porcine aortas were purchased from Chenggong slaughterhouse in Kunming. The geometry of porcine arteries was as close as possible to that of human thoracic arteries. The geometric dimensions of human (Labrosse et al., 2013; Mensel et al., 2014; Mensel et al., 2016) and porcine arterial vessels are listed in Table S1 in Supplementary Material. Collection of and experiments on porcine artery tissues were approved by the Ethics Committee of Kunming University of Science and Technology.

#### 2.2.2 Simulation device of arterial vascular aging

Figure S1 in Supplementary Material shows the arterial aging simulation device.

#### 2.2.3 Arterial aging simulation experiments

The experiment aimed to obtain the changes in porcine aortic geometry of inner, outer diameter and thickness and expansion curves under different AAPSRs to calculate the strain energy of the elastic arteries. Constraints were applied to the vessel surface to simulate the constraint conditions of living soft tissues (Guo et al., 2016). The treated porcine aorta was fixed on the experimental platform and its inner diameter, outer diameter, and wall thickness were measured. The initial AAPSR value was 1.33, which decreased gradually to 1.01. The outer diameter of the aorta was measured using a laser rangefinder (Mitutoyo LUMS-503S, United States). Because the normal blood pressure of pigs is 113/60 mmHg and their heart rate is 80 bpm (Gladczak et al., 2013; Lelovas et al., 2014), the pressure range of the device (Electroforce 5500, TA Instruments, United States) was 60–120 mmHg. To mimic the changes in the AAPSR during human aging, the AAPSRs were scaled from high to low at 1.33, 1.23, 1.08, 1.05, and 1.01 in the stretch-inflation tests. The effects of vascular lesions were excluded from this experiment.

### 2.3 Stiffness and circumferential elastic modulus

Based on the definition of compliance, the aortic compliance was calculated as follows:

$$C=\frac{∆V}{∆P}=\frac{\pi *l*\left(D_{s}^{2}-D_{d}^{2}\right)}{4*PP}$$ (9)

where *C* is aortic compliance, l is aortic unit length, and PP is pulse pressure, $PP=p_{s}-p_{d}$ . Owing to its geometric dimensions, the aorta can be considered as a thin-walled tube. The equations for circumferential stress and strain are $\sigma_{\theta }=\varphi DP/2h$ and $\varepsilon_{\theta }=\left(D_{s}-D_{o}\right)/D_{o}\;-\left(D_{d}-D_{o}\right)/D_{o}$ respectively. The circumferential elastic modulus can be given by

$$E=\frac{DPD_{o}}{2h\left(D_{s}-D_{d}\right)}$$ (10)

where $D_{o}$ is the initial internal diameter of the aorta.

### 2.4 Statistics

All the data in this study are recorded as mean ± standard error.

## 3 Results

### 3.1 Thoracic artery compliance and elastic modulus of porcine

**FIGURE 2:**
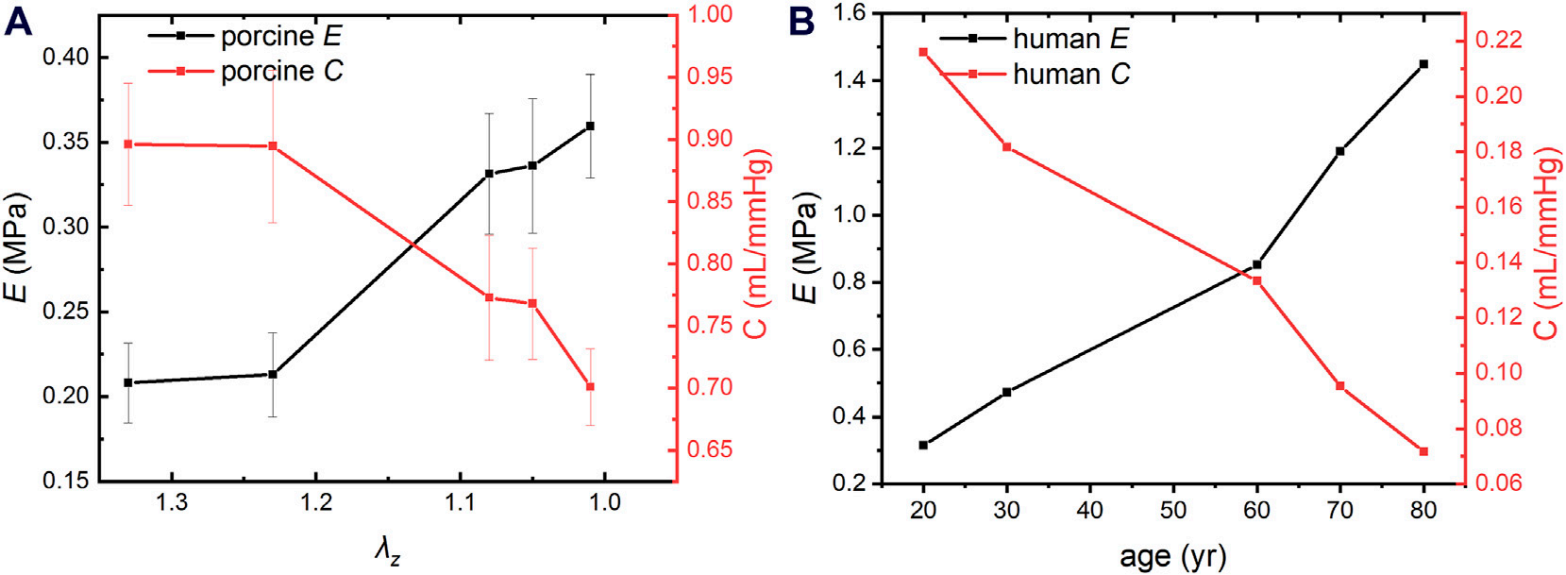
Compliance and circumferential elastic modulus of porcine and human aortas. **(A)** Compliance and circumferential elastic modulus of porcine aorta. **(B)** Compliance and circumferential elastic modulus of human aorta.

Aortic compliance and circumferential elastic modulus are biomarkers of vascular elasticity. The arterial compliance and circumferential elastic modulus of porcine aortas were calculated using Eqs 9, 10, as shown in Figure 2A. Aortic compliance decreases and the circumferential elastic modulus increases with the decrease of AAPSR. The circumferential elastic modulus and flexibility of porcine arteries varied little between *λ*_*z*_ of 1.33 and 1.23, more between 1.23∼1.08, and the greatest change rate was between 1.08–1.01. This trend is consistent with changes in human arteries (Mikael et al., 2017; Jadidi et al., 2020).

### 3.2 SEDI in elastic arteries

**FIGURE 3:**
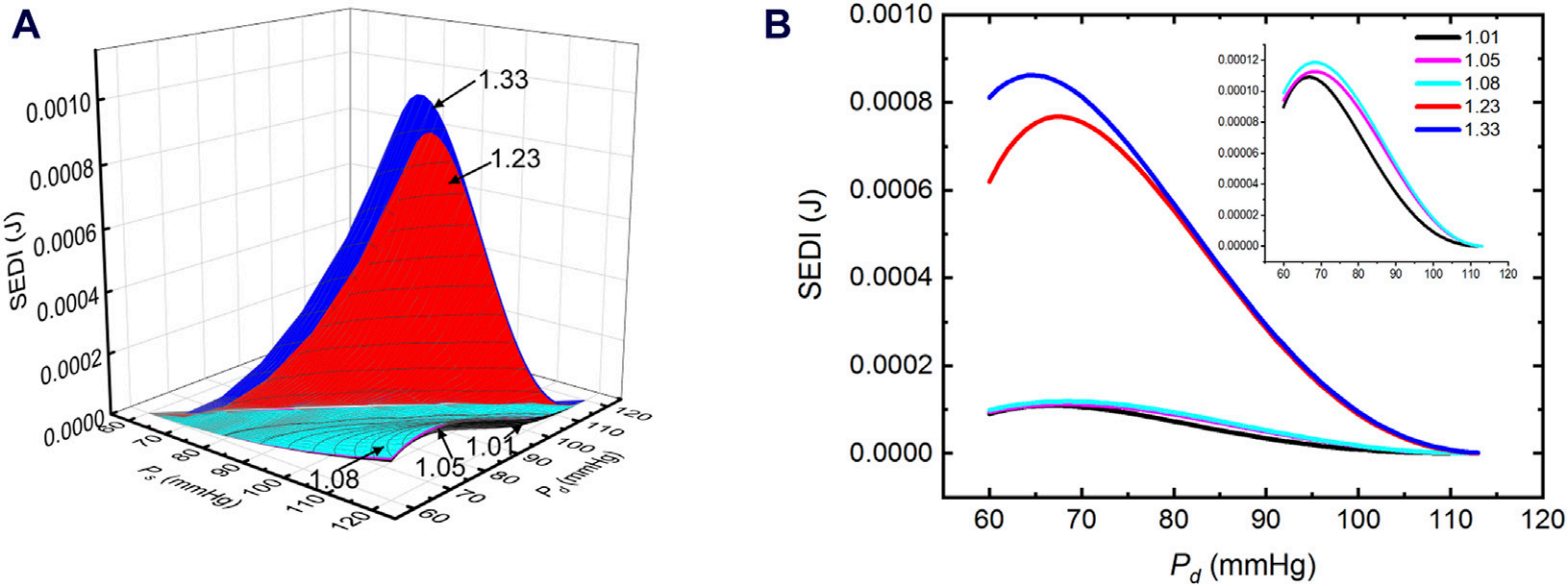
SEDI of porcine thoracic aorta; **(A)** Three-dimensional SEDI of porcine thoracic aortas. **(B)** SEDI of the thoracic aorta at a systolic blood pressure ( $P_{s}$ ) of 113 mmHg.

The promotion of blood flow through the elastic arteries is a natural phenomenon. To the best of our knowledge, only a few studies have quantified the contribution of elastic vessels to blood flow. The contribution of elastic vessels to blood flow is mainly manifested in the storage of strain energy in deformed vessels *in vivo*, which is converted into mechanical energy to promote blood flow (Cheng et al., 2022). The SEDI of the elastic arteries of the porcine aorta under different diastolic and systolic pressures and at different AAPSRs were calculated according to Eq. 1, as shown in Figure 3. Our results show that pre-stretched arterial vessel stores strain potential energy. The SEDI reached its maximum value under appropriate systolic and diastolic pressure combinations and varied with the AAPSR. The strain energy reached its maximum value when the AAPSR value is between 1.33∼1.23, whereas hardly changed between 1.23∼1.08. The change in SEDI reached maximum with AAPSR value of 1.23∼1.08, the change was stable and reached minimum when AAPSR was between 1.08∼1.01. The maximum SEDI of *λ*_*z*_ = 1.01 decreased by 87.21% compared to that of 1.33. The most changes of SEDI with AAPSR occurred within the lower diastolic BP ranges with aging. However, all ages had lower SEDI when diastolic BP was heightened >80 mmHg which is the clinically relevant cut off for prehypertension.

### 3.3 Elastic modulus and compliance of human thoracic arteries

The arterial compliance and circumferential elastic modulus of human thoracic arteries were calculated based on human anatomical data and Eqs 9, 10, as shown in Figure 2B. The modulus of elasticity of the human thoracic artery increased by an average of 0.0134 MPa per year between 20 and 60 years of age and 0.0298 MPa per year between 60 and 80 years of age, indicating that arterial vascular stiffness increases more rapidly in the older adults and accelerated aging. Notably, the modulus of elasticity is much smaller in pigs than in humans. This is because human vascular data were collected from many volunteers rather than from an individual’s arteries, and human blood vessels contain vascular lesions. However, both trends are consistent with clinical statistical results (Mikael et al., 2017; Jadidi et al., 2020).

### 3.4 Calculation of mean blood flow through human thoracic artery

**FIGURE 4:**
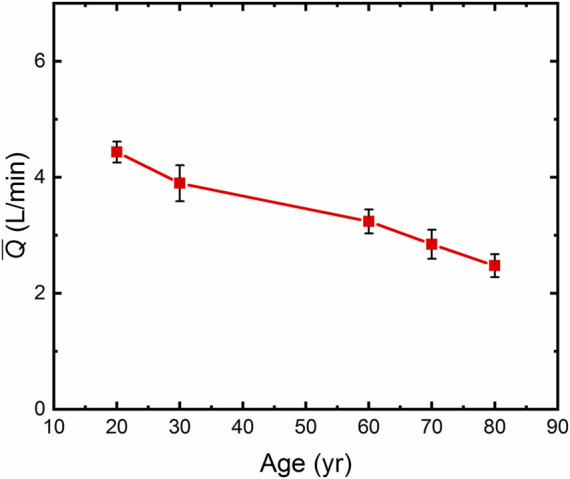
The Average thoracic artery flow ( $\bar{Q}$ ) in humans by age.

The mean blood flow was calculated according to Eq. 8, based on the human vascular anatomical data and age-related blood rheological properties (Cinelli et al., 1987; Coppola et al., 2000; Labrosse et al., 2009; Wilkins et al., 2010; Horny et al., 2014b; Conen et al., 2014; Tarumi et al., 2014; Jadidi et al., 2020). The structural and functional parameters of the human thoracic arteries are shown in Supplementary Table S1, and the thoracic arterial blood supply was calculated for different ages of human, as shown in Figure 4. The results showed that the blood flow decreased with the increase of age. In humans, the average annual decrease in blood supply was 0.0298 L/min for those aged 20∼60 years old, and 0.0383 L/min for 60∼80 years old. The results show that SP decreases with age, which is one of the manifestations of the occurrence of chronic diseases in the elderly. Human blood flow was 44.2% lower at the age of 80 than that at the age of 20, which is consistent with clinical results (Brandfonbrener et al., 1955).

### 3.5 Calculation of SP and CBF with OH occurrence

The blood flow through the ascending arteries, that is, the SP, was calculated using Eq. 8 based on the different classifications of OH for the 60 years old with the baseline BP of 129.1/79.8 mmHg (SBP/DBP) (Conen et al., 2014), and for the 80 years old with the baseline BP of 154/76 mmHg (Abad Pérez et al., 2022), and the results were listed in Table 1. It is well known that the carotid arteries are extremely high-flow vessels, and in the elderly population, approximately 12∼15% of the SP is mainly delivered to the brain through these vessels with a diameter of 4∼5 mm (Xing et al., 2017). As shown in Table 1, a decrease in BP had a significant effect on SP in the presence of OH.

**TABLE 1:** Calculation of blood flow through the ascending artery.

	Baseline-60 years	OH					Baseline-80 years	Hypertension in older adults-OH-80 years						
*P*_*s*_ (mmHg)	129.1	119.1	109.1	99.1	109.1	99.1	154	144	134	124	114	134	124	114
*P*_*d*_ (mmHg)	79.8	79.8	79.8	79.8	69.8	69.8	76	76	76	76	76	66	66	66
*P* (mmHg)	96.2	92.9	89.6	86.2	82.9	79.6	102	98.7	95.3	92	88.7	88.7	85.3	82
*λ*_*z*_	1.08	1.08	1.08	1.08	1.08	1.08	1.01	1.01	1.01	1.01	1.01	1.01	1.01	1.01
*ε*_*Ap*_	0.015	0.015	0.015	0.015	0.015	0.015	0.01	0.01	0.01	0.01	0.01	0.01	0.01	0.01
*D*_*so*_ (mm)	35.4	35.4	35.4	35.4	35.4	35.4	35.9	35.9	35.9	35.9	35.9	35.9	35.9	35.9
*h*_*s*_ (mm)	1.41	1.41	1.41	1.41	1.41	1.41	1.42	1.42	1.42	1.42	1.42	1.42	1.42	1.42
*D*_*s*_ (mm)	32.6	32.6	32.6	32.6	32.6	32.6	33.1	33.1	33.1	33.1	33.1	33.1	33.1	33.1
*α*_*s*_	23.1	23.1	23.1	23.1	23.1	23.1	23.3	23.3	23.3	23.3	23.3	23.3	23.3	23.3
*D*_*do*_ (mm)	34.32	34.32	34.32	34.32	34.32	34.32	33.92	33.92	33.92	33.92	33.92	33.92	33.92	33.92
*h*_*d*_ (mm)	1.46	1.46	1.46	1.46	1.46	1.46	1.51	1.51	1.51	1.51	1.51	1.51	1.51	1.51
*D*_*d*_ (mm)	31.4	31.4	31.4	31.4	31.4	31.4	30.9	30.9	30.9	30.9	30.9	30.9	30.9	30.9
*α*_*d*_	21.5	21.5	21.5	21.5	21.5	21.5	20.5	20.5	20.5	20.5	20.5	20.5	20.5	20.5
*E* (MPa)	0.7	0.7	0.7	0.7	0.7	0.7	1.4	1.4	1.4	1.4	1.4	1.4	1.4	1.4
*φ*	0.038	0.0435	0.0435	0.0435	0.0435	0.0435	0.0425	0.0425	0.0425	0.0425	0.0425	0.0425	0.0425	0.0425
*Re*	909.09	909.09	909.09	909.09	909.09	909.09	833	833	833	833	833	833	833	833
*β*	1.24	1.24	1.24	1.24	1.24	1.24	2.91	3.01	3.01	3.01	3.01	3.01	3.01	3.01
*V* (m)	0.123	0.119	0.104	0.088	0.118	0.104	0.119	0.111	0.103	0.096	0.087	0.110	0.103	0.094
*Q* (L/bpm)	0.096	0.093	0.082	0.069	0.092	0.081	0.09	0.084	0.078	0.072	0.066	0.083	0.078	0.072
*n* (bpm)	66.1	66.1	66.1	66.1	66.1	66.1	67	67	67	67	67	67	67	67
$\bar{Q}$ (L/min)	6.37	6.16	5.43	4.58	6.11	5.38	6.06	5.62	5.25	4.84	4.41	5.58	5.20	4.79
CBF (mL/min)	765∼956	739∼924	652∼814	550∼688	733∼917	645∼806	727∼908	675∼844	630∼787	581∼727	528∼660	670∼837	624∼780	575∼719
△(%)		−3.33	−14.79	−28.05	−4.08	−15.65		−7.11	−13.32	−20.01	−27.32	−7.86	−14.12	−20.88

Note: *P*_*s*_ and *P*_*d*_ denote the systolic and diastolic blood pressure, *P* denotes the mean blood pressure, *λ*_*z*_ means AAPSR, *ε*_*Ap*_ denotes strain of the cross section area, *D*_*so*_, *D*_*s*_ and *h*_*s*_ denote outer and inner diameter and artery wall thickness during the systolic period, *D*_*do*_, *D*_*do*_ and *h*_*s*_ denote outer and inner diameter and artery wall thickness during the diastolic period, *α*_*s*_ = *D*_*s*_ */h*_*s*_, *α*_*d*_ = *D*_*d*_ */h*_*d*_, *E* means circumferential elastic modulus, *φ* means the constraint coefficient of the aorta, *Re* is the Reynolds number, *β* is the coupled energy dissipation coefficient, *V* means the characteristic velocity, *Q* means the blood through the cross-section per stroke, *n* is the pulse frequency, $\bar{Q}$ mean the blood flow per minute, CBF denotes the cerebral perfusion, CBF = $\bar{Q}$ *12%∼15%, △ denotes change rate of blood flow.

As can be seen in Table 1, compared to the baseline state of normal 60-year-old population, when SBP decreased by 20 mmHg and 30 mmHg, SP reduced by 14.79% and 28.05%, respectively, and when SBP decreased by 30 mmHg and DBP decreased by 10 mmHg, SP reduced by 15.65%. When SBP decreased by 20, 30 and 40 mmHg, SP decreased by 13.32%, 20.01% and 27.32% respectively. When SBP decreased by 30 mmHg and DBP decreased by 10 mmHg, SP decreased by 20.88% in 80-year-old hypertension patients, correspondingly, CBF reduced by 12∼15%.

## 4 Discussion

The development of cognitive dysfunction and dementia is a complex, multifactorial process. Chronic hypoperfusion of the CBF is an important factor leading to cognitive decline and accelerated neurodegeneration (Claassen et al., 2021). Based on this mechanistic hypothesis, the relationship between SP, CBF, and OH symptoms will be discussed in the following context, which in turn can explain: the close correlation between OH, CF decline and dementia and provide a basis for the increased odds of CI and dementia with the increased occurrence of OH. OH is associated with an increased risk of cardiovascular and cerebrovascular diseases and all-cause mortality.

### 4.1 OH and SP and CBF

OH is closely related to SP and CBF. In general, the geometric parameters of the arterial vasculatures and rheological parameters of the blood do not change abruptly during the onset of OH. The main symptom of OH is a transient change in vascular functional parameters (i.e., SBP, DBP, and PP), which can be attribute to autonomic nervous system dysfunction. As shown in Table 1; Eq. 8, the SP changes with instantaneous and abrupt changes in BP. SP varies under different BP levels, and so does the corresponding CBF. In a clinical context, acute reductions in global CBF can occur with cardiac arrest (Lipton, 1999) or with a sudden profound reduction in BP, which can lead to global reductions in CBF, termed syncope (Claassen et al., 2021). When the CBF exceeds the normal threshold, it causes damage to brain cells, especially when the cerebral vessels are chronically hypoperfused. There are no exact thresholds beyond which ischemic injury occurs, but estimates are that a sustained fall to 20%–30% of baseline CBF causes ischemia within minutes (Astrup et al., 1981; Siesjö, 1992; Lipton, 1999). In addition, SP decreases with age and arterial vascular aging (see Figure 4). Decreased SP affects cerebral perfusion and leads to a common phenomenon of mixed pathology. OH is closely associated with decreased cognitive function and dementia, particularly in AD in the elderly, vascular dementia and mixed senile dementia. Min et al. researched the association between OH and dementia using meta-analysis of prospective cohort study, 22.4% higher prevalence of dementia in OH subjects (hazard ratio (HR): 1.224; 95% CI: 1.106–1.354; *p* < 0.001). This meta-analysis also showed significant association between OH and two dementia subtypes: AD (HR: 1.175; 95% CI: 1.022–1.351; *p* = 0.023) and vascular dementia (HR: 1.403; 95% CI: 1.042–1.889; *p* = 0.026), respectively (Min et al., 2018). Rawlings et al. analyzed 11,503 participants and found persons with OH were 40% more likely to develop dementia than those without OH (HR: 1.40, 95%, CI: 1.13, 1.73). Persons with OH had significantly more cognitive decline over 20 years compared to those without (difference: −0.12, 95% CI: −0.23, −0.02) (Rawlings et al., 2017). This finding is also consistent with clinical studies (Glodzik et al., 2019; Isik et al., 2020; Singh and Cheng, 2021). Eq. 8 confirmed that the hypothesized mechanism of long-term CBF in the state of low perfusion is the cause of cognitive decline and accelerated neurodegeneration.

### 4.2 OH and SBP

This study also shown that SBP regulated SP and CBF is more strongly associated with OH occurrence than that of DBP. In the general population, occasional OH is not sufficient to cause damage to brain cells, however, the frequency of OH increases with age, and prolonged and repeated OH can lead to chronic cerebral hypoperfusion, which increases the odds of cognitive impairment and dementia. However, when OH occurs, SBP regulates SP and CBF to a greater extent than DBP does. Thus, when OH occurs, SBP regulation is more strongly associated with CI and dementia than DBP regulation. More severe OH is associated with greater risk. The magnitude of the SBP decline while standing up is associated with the risk of dementia. Rouch et al. examined systolic OH and diastolic OH of 2,131 older adults separately, the results showed that, unlike diastolic OH, systolic OH was associated with greater dementia risk (HR = 1.37, 95% CI: 1.01–1.88). SBP postural changes variability was also associated with higher dementia risk (highest coefficient of variation, HR = 1.35, 95% CI: 1.06–1.71) (Rouch et al., 2020). This is consistent with the findings of Hilkens et al. (2021) (Robertson et al., 2019; Rouch et al., 2020). Table 1 identifies that the changes in CBF was strongly dependent on the speed of BP changes (Birch et al., 1995). The slower the change in BP, the smaller the impact on CBF, to a point where CBF becomes almost unaffected. However, for more rapid changes in BP, changes in CBF become larger, until a point where changes in CBF become as large as the change in BP. At that point, CBF passively follows BP. This is consistent with the findings of Birch et al. (1995).

### 4.3 OH and CCVD

OH is associated with increased risk of cardio-cerebrovascular disease (CCVD) and all-cause mortality. Prolonged and sustained OH leads to a chronic ischemic state in systemic organs and tissues, and the autonomic nervous system compensates for and maintains SP and CBF by increasing BP or PP. As can be seen from Eq. 8; Table 1; Figure 4, the SP and CBF is reduced owing to OH. In addition, with the increase of aging, arterial vascular stiffness increases gradually (see Figure 2), and the strain energy that drives blood flow decreases, increasing the risk of CCVD and all-cause mortality. Alicia et al. researched a total of 10,611 subjects, 11 studies were included, the results showed that increased arterial stiffness raises the risk of OH (odds ratio: 1.40, 95% CI: 1.28–1.54), with a stronger association with central arterial stiffness (odds ratio: 1.50, 95% CI: 1.34–1.68) than with peripheral arterial stiffness (odds ratio: 1.29, 95% CI: 1.17–1.43) (Saz-Lara et al., 2023). Xia et al. conducted a 15-year population-based cohort study included 2,703 dementia-free participants (mean age at baseline, 73.7 years) who were divided into the CVD-free cohort (N = 1986) and the CVD cohort (N = 717). OH was associated with CVD with the hazard ratio of 1.33 (95% CI: 1.12–1.59). OH was not significantly associated with incident dementia in the absence of CVD occurring before dementia diagnosis (HR: 1.22, 95% CI: 0.83–1.81). In the CVD cohort, individuals with OH had a higher dementia risk than those without OH (HR: 1.54, 95% CI: 1.06–2.23) (Xia et al., 2023). The prevalence of OH is 50% higher in patients with hypertension than adults with normal BP (Frewen et al., 2014), whereas patients with mild dementia have a higher prevalence of OH (Sonnesyn et al., 2009), sustained time of low SP and CBF will also be lengthen. Sustained decreased SP leads to an increased risk of CCVD such as coronary heart disease, heart attack, and cerebral infarction. Thus, OH is associated with an increased risk of CCVD and all-cause mortality. Appropriate BP control is reasonable for the prevention of dementia. A sustained reduction in SBP on standing up is an independent risk factor for death with a 45% 5-year mortality (Frith et al., 2016).

### 4.4 Slight and sudden BP drop

The risk of CI may also be elevated even with a slight drop in BP. Table 1 shows that compared to the baseline state of 60-year-old healthy people SP decreased by 3.33% when SBP decreased by 10 mmHg, accordingly, SP decreased by 7.11%, CBF decreased in 80-year-old patients with hypertension. In other words, SP and CBF are reduced even with a sudden and slight decrease in BP, which does not satisfy the classical OH criteria. It can also be seen from Figures 2–4 that with an increase of age, human arterial AAPSR value decreases, arterial stiffness increases, reducing the strain energy to propel blood flow, resulting in a corresponding decrease in the total blood supply. Both may occur simultaneously, or either occurs solely, and the interconnection of these risk factors results in chronically hypoperfused CBF (Angoff et al., 2021). Therefore, the risk of CI elevated with a sudden slight drop in BP, the only difference is in the course of CI development. Of cause SP decrease would be greater when BP drops > 20 mmHg, which would be more harmful and have clinical relevance.

CI also occurs in people without OH, and the prevalence of OH is significantly higher in patients with dementia than in those without it (Hayakawa et al., 2015), and low BP in patients with CI is more likely to convert to dementia (Guo et al., 1996).

### 4.5 OH and carotid artery stenosis

Greater risk when coexists of OH and carotid artery stenosis as evidence suggests that in carotid atherosclerotic diseases cerebral atrophy and vascular cognitive impairment may be associated (Droste et al., 1996; Siebler et al., 1996). Carotid atherosclerosis and plaques cause various types of cognitive dysfunctions and are common in asymptomatic individuals. Inadequate cerebral perfusion is believed to accelerate amyloid and tau protein deposition, which is a potential link between limited blood flow and cognitive dysfunction (Daulatzai, 2017). However, few studies have evaluated the association between plaque formation and development of cognitive dysfunction and dementia. Current literature shows inconsistent results regarding the association between vulnerable plaque components and cognitive dysfunction. As can be seen from Eq. 8; Table 2, CBF decreases with the decrease of overflow area *A*_*f*_. The blockage of the internal carotid arteries is a progressive process. Therefore, cerebral ischemia is a progressive process. When OH coexists with carotid artery stenosis, it accelerates the progression of cerebral infarction, cognitive decline, and dementia. In one of the largest studies evaluating this association in over 4,000 participants, the authors found that high-grade carotid artery stenosis was associated with cognitive impairment (odds ratio: 6.7, 95% CI: 2.4–18.1) and cognitive decline (odds ratio: 2.6, CI: 1.1–6.3) (Baradaran et al., 2021). Khan et al. shown that almost 50% of patients with asymptomatic carotid stenosis would exhibit cognitive impairment. Some studies have also shown that the asymptomatic carotid stenosis could results in cerebral hypoperfusion. Out of 20 patients, 18 had unilateral stenosis (8 right and 10 left) and 2 had bilateral stenosis. The interhemispheric (left–right) time to peak delays measured for the whole brain volume identified impaired perfusion in the hemisphere ipsilateral to the stenosis in 16 of the 18 patients. More than 45% of the patients had ischemia in at least one half of their brain volume (Khan et al., 2021). Formula 8 is consistent with these above clinical results, and the CBF of individuals is accurately calculated with Formula 8.

**TABLE 2:** Calculated values of CBF with different degree of stenosis (Arnett et al., 2000; Gundogan et al., 2013; Nie et al., 2022) in the carotid artery.

Degree of stenosis	0%	50 (%)	70 (%)	80 (%)	90 (%)
*A*_*f*_ */A*_*fo*_	100%	50%	37.4	31.2	25.2
△CBF (%)	0	−41.8	−51.0	−55.2	−61.8

Note: *A*_*f*_-denotes blood flow area through the aorta, *A*_*fo*_-denotes cross sectional area of carotid artery without stenosis, △CBF denotes the change ratio of CBF with and without stenosis.

### 4.6 OH in older patients with hypertension and cognitive decline or dementia

The etiology of OH in the general population is predominantly non-autonomic, and autonomic dysfunction induces OH in a bidirectional way. The prevalence of OH is 50% higher in hypertension patients than adults with normal BP (Frewen et al., 2014), and patients with hypertension are at greater risk of cognitive decline or dementia. As can be seen in Table 1, for the 80-years old group, compared with the 60-years-old normal BP group, when SBP decreased by 20, 30 and 40 mmHg, SP decreased by 17.58%, 24.02% and 30.77%, respectively. Correspondingly, CBF reduces by 12%–15% of systemic perfusion. The prevalence of OH increased from 9.4% to 14.1% by age increased from 70 years to 85 years old (Rutan et al., 1992). In the Kungsholmen Project, a cohort of 1,270 individuals (aged ≥ 75 years) were followed during 6 years. The results revealed that individuals with higher SBP (>180 mmHg) had a relative risk of 1.5 for AD (95% CI: 1.0–2.3), and 1.6 for dementia (95% CI: 1.1–2.2) in general (Qiu et al., 2003). One study (382 individuals aged 70 years; followed 15 years) showed a relationship between both increased SBP and DBP and the diagnostic of AD or dementia later. Results showed that individuals who developed dementia 15 years later had higher SBP and DBP values at baseline (70 years old) compared to individuals without dementia (Skoog et al., 1996). This indicated that the risk of cognitive decline or dementia is much higher in older patients with hypertension than in the general population. In individuals with cognitive dysfunction, CBF is in the state of low perfusion, and OH exacerbates its transformation into dementia.

In Conclusion, the proposed fluid-structure interaction model of blood circulation can quantitatively analyze the relationship between SP and CBF when OH occurs, and we hope to contribute to the field of clinical research on OH with CI and dementia and promote the progress of this topic. The findings suggested that OH is strongly associated with SP and CBF; SBP regulation is more strongly associated with SP and CBF than DBP does during the occurrence of OH; OH is associated with an increased risk of CCVD and all-cause mortality, with the increase in age of aging and arterial vasculopathy, even with a sudden and slight decrease in BP, the risk of CI and dementia will be elevated; and, in the elderly group, the coexistences of OH and arterial stenosis indicates a greater risk.

## 5 Limitations

The present study only considered alterations in SP and CBF when OH occurs in the older population and did not consider the effects of other dementia risk factors.

## Data Availability

The original contributions presented in the study are included in the article/Supplementary Material, further inquiries can be directed to the corresponding authors.

## References

[B1] Abad Pérez D. García Polo I. Rodríguez Salvanés F. J. Bellisco Roncal S. Ibáñez Sanz P. Suárez Fernández C. (2022). Sustained-release isosorbide mononitrate as adjuvant treatment in isolated systolic hypertension in the elderly. J. Hum. Hypertens. 36 (2), 163–170. 10.1038/s41371-021-00498-4 33850272

[B2] Angelousi A. Girerd N. Benetos A. Frimat L. Gautier S. Weryha G. (2014). Association between orthostatic hypotension and cardiovascular risk, cerebrovascular risk, cognitive decline and falls as well as overall mortality: a systematic review and meta-analysis. J. Hypertens. 32 (8), 1562–1571. 10.1097/hjh.0000000000000235 24879490

[B3] Angoff R. Mosarla R. C. Tsao C. W. (2021). Aortic stiffness: epidemiology, risk factors, and relevant biomarkers. Front. Cardiovasc Med. 8, 709396. 10.3389/fcvm.2021.709396 34820427 PMC8606645

[B4] Arnett D. K. Boland L. L. Evans G. W. Riley W. Barnes R. Tyroler H. A. (2000). Hypertension and arterial stiffness: the atherosclerosis risk in communities study. ARIC investigators. Am. J. Hypertens. 13, 317–323. 10.1016/s0895-7061(99)00281-2 10821330

[B5] Astrup J. Siesjö B. K. Symon L. (1981). Thresholds in cerebral ischemia - the ischemic penumbra. Stroke 12 (6), 723–725. 10.1161/01.str.12.6.723 6272455

[B6] Baradaran H. Sarrami A. H. Gupta A. (2021). Asymptomatic carotid disease and cognitive impairment: what is the evidence? Front. Neurol. 12, 741500. 10.3389/fneur.2021.741500 34867724 PMC8636319

[B7] Birch A. A. Dirnhuber M. J. Hartley-Davies R. Iannotti F. Neil-Dwyer G. (1995). Assessment of autoregulation by means of periodic changes in blood pressure. Stroke 26 (5), 834–837. 10.1161/01.str.26.5.834 7740576

[B8] Brandfonbrener M. Landowne M. Shock N. W. (1955). Changes in cardiac output with age. Circulation 12 (4), 557–566. 10.1161/01.cir.12.4.557 13261308

[B9] Cheng H. Li G. Dai J. Zhang K. Xu T. Wei L. (2022). A fluid-structure interaction model accounting arterial vessels as a key part of the blood-flow engine for the analysis of cardiovascular diseases. Front. Bioeng. Biotechnol. 10, 981187. 10.3389/fbioe.2022.981187 36061431 PMC9438578

[B10] Cicconetti P. Riolo N. Priami C. Tafaro L. Ettore E. (2004). Risk factors for cognitive impairment. Recenti Prog. Med. 95 (11), 535–545. 15598092

[B11] Cinelli P. de Leonardis V. De Scalzi M. Becucci A. Grazzini M. (1987). Effect of age on mean heart rate and heart rate variability. Age 10 (4), 146–148. 10.1007/BF02432162

[B12] Claassen J. Thijssen D. H. J. Panerai R. B. Faraci F. M. (2021). Regulation of cerebral blood flow in humans: physiology and clinical implications of autoregulation. Physiol. Rev. 101 (4), 1487–1559. 10.1152/physrev.00022.2020 33769101 PMC8576366

[B13] Coccarelli A. Hasan H. M. Carson J. Parthimos D. Nithiarasu P. (2018). Influence of ageing on human body blood flow and heat transfer: a detailed computational modelling study. Int. J. Numer. methods Biomed. Eng. 34 (10), e3120. 10.1002/cnm.3120 PMC6220937 29932495

[B14] Conen D. Aeschbacher S. Thijs L. Li Y. Boggia J. Asayama K. (2014). Age-specific differences between conventional and ambulatory daytime blood pressure values. Hypertension 64 (5), 1073–1079. 10.1161/HYPERTENSIONAHA.114.03957 25185130 PMC11006446

[B15] Coppola L. Caserta F. De Lucia D. Guastafierro S. Grassia A. Coppola A. (2000). Blood viscosity and aging. Archives Gerontology Geriatrics 31 (1), 35–42. 10.1016/s0167-4943(00)00063-7 10989162

[B16] Cremer A. Soumaré A. Berr C. Dartigues J. F. Gabelle A. Gosse P. (2017). Orthostatic hypotension and risk of incident dementia: results from a 12-year follow-up of the three-city study cohort. Hypertension 70 (1), 44–49. 10.1161/hypertensionaha.117.09048 28559394

[B17] Daulatzai M. A. (2017). Cerebral hypoperfusion and glucose hypometabolism: key pathophysiological modulators promote neurodegeneration, cognitive impairment, and Alzheimer’s disease. J. Neurosci. Res. 95 (4), 943–972. 10.1002/jnr.23777 27350397

[B18] Droste D. W. Decker W. Siemens H. J. Kaps M. Schulte-Altedorneburg G. (1996). Variability in occurrence of embolic signals in long term transcranial Doppler recordings. Neurol. Res. 18 (1), 25–30. 10.1080/01616412.1996.11740372 8714532

[B19] Elmstáhl S. Rosén I. (1997). Postural hypotension and EEG variables predict cognitive decline: results from a 5-year follow-up of healthy elderly women. Dement. Geriatr. Cogn. Disord. 8 (3), 180–187. 10.1159/000106629 9137897

[B20] Freis E. D. Heath W. C. (1964). Hydrodynamics of aortic blood flow. Circ. Res. 14, 105–116. 10.1161/01.res.14.2.105 14118756

[B21] Frewen J. Finucane C. Savva G. M. Boyle G. Kenny R. A. (2014). Orthostatic hypotension is associated with lower cognitive performance in adults aged 50 plus with supine hypertension. J. Gerontol. A Biol. Sci. Med. Sci. 69 (7), 878–885. 10.1093/gerona/glt171 24214492

[B22] Frith J. Bashir A. S. Newton J. L. (2016). The duration of the orthostatic blood pressure drop is predictive of death. Qjm 109 (4), 231–235. 10.1093/qjmed/hcv126 26163077

[B23] Gladczak A. K. Shires P. K. Stevens K. A. Clymer J. W. (2013). Comparison of indirect and direct blood pressure monitoring in normotensive swine. Res. Veterinary Sci. 95 (2), 699–702. 10.1016/j.rvsc.2013.05.013 23790711

[B24] Glodzik L. Rusinek H. Tsui W. Pirraglia E. Kim H. J. Deshpande A. (2019). Different relationship between systolic blood pressure and cerebral perfusion in subjects with and without hypertension. Hypertension 73 (1), 197–205. 10.1161/hypertensionaha.118.11233 30571554 PMC7986962

[B25] Gundogan F. C. Colakoglu K. Isilak Z. (2013). Cone pathway function in relation to asymmetric carotid artery stenosis: correlation to blood pressure. Acta Ophthalmol. 91 (1), e71. 10.1111/j.1755-3768.2012.02567.x 23210641

[B26] Guo J. P. Jia X. Sai Z. Ge Y. Y. Wang S. Guo W. (2016). Thoracic aorta dimension changes during systole and diastole: evaluation with ECG-gated computed tomography. Ann. Vasc. Surg. 35, 168–173. 10.1016/j.avsg.2016.01.050 27263817

[B27] Guo Z. Viitanen M. Fratiglioni L. Winblad B. (1996). Low blood pressure and dementia in elderly people: the Kungsholmen project. B M. J. 312 (7034), 805–808. 10.1136/bmj.312.7034.805 PMC2350725 8608286

[B28] Hayakawa T. McGarrigle C. A. Coen R. F. Soraghan C. J. Foran T. Lawlor B. A. (2015). Orthostatic blood pressure behavior in people with mild cognitive impairment predicts conversion to dementia. J. Am. Geriatr. Soc. 63 (9), 1868–1873. 10.1111/jgs.13596 26313614

[B29] Hayashida K. Nishiooeda Y. Hirose Y. Ishida Y. Nishimura T. (1996). Maladaptation of vascular response in frontal area of patients with orthostatic hypotension. J. Nucl. Med. 37 (1), 1–4. 8543976

[B30] Hebert L. E. Weuve J. Scherr P. A. Evans D. A. (2013). Alzheimer disease in the United States (2010-2050) estimated using the 2010 census. Neurology 80 (19), 1778–1783. 10.1212/WNL.0b013e31828726f5 23390181 PMC3719424

[B31] Hilkens N. A. Klijn C. J. M. Richard E. (2021). Blood pressure, blood pressure variability and the risk of poststroke dementia. J. Hypertens. 39 (9), 1859–1864. 10.1097/hjh.0000000000002841 33710171

[B32] Horny L. Adamek T. Gultova E. Zitny R. Vesely J. Chlup H. (2011). Correlations between age, prestrain, diameter and atherosclerosis in the male abdominal aorta. J. Mech. Behav. Biomed. Mater 4 (8), 2128–2132. 10.1016/j.jmbbm.2011.07.011 22098912

[B33] Horny L. Adamek T. Kulvajtova M. (2014a). Analysis of axial prestretch in the abdominal aorta with reference to post mortem interval and degree of atherosclerosis. J. Mech. Behav. Biomed. Mater 33, 93–98. 10.1016/j.jmbbm.2013.01.033 23676503

[B34] Horny L. Netusil M. Vonavkova T. (2014b). Axial prestretch and circumferential distensibility in biomechanics of abdominal aorta. Biomech. Model Mechanobiol. 13 (4), 783–799. 10.1007/s10237-013-0534-8 24136338

[B35] Hughes D. Judge C. Murphy R. Loughlin E. Costello M. Whiteley W. (2020). Association of blood pressure lowering with incident dementia or cognitive impairment: a systematic review and meta-analysis. Jama 323 (19), 1934–1944. 10.1001/jama.2020.4249 32427305 PMC7237983

[B36] Isik A. T. Kocyigit S. E. Kaya D. Dost Gunay F. S. Erken N. Dokuzlar O. (2020). The relationship between the most common subtypes of dementia and orthostatic hypotension in older adults. Dement. Geriatr. Cogn. Disord. 49 (6), 628–635. 10.1159/000513978 33735870

[B37] Isik A. T. Kocyigit S. E. Smith L. Aydin A. E. Soysal P. (2019). A comparison of the prevalence of orthostatic hypotension between older patients with Alzheimer’s Disease, Lewy body dementia, and without dementia. Exp. Gerontol. 124, 110628. 10.1016/j.exger.2019.06.001 31173842

[B38] Jadidi M. Habibnezhad M. Anttila E. Maleckis K. Desyatova A. MacTaggart J. (2020). Mechanical and structural changes in human thoracic aortas with age. Acta Biomater. 103, 172–188. 10.1016/j.actbio.2019.12.024 31877371 PMC6982607

[B39] Juraschek S. P. Daya N. Appel L. J. Miller E. R. McEvoy J. W. Matsushita K. (2018). Orthostatic hypotension and risk of clinical and subclinical cardiovascular disease in middle-aged adults. J. Am. Heart Assoc. 7 (10), e008884. 10.1161/jaha.118.008884 29735525 PMC6015335

[B40] Kamenskiy A. Seas A. Bowen G. Deegan P. Desyatova A. Bohlim N. (2016). *In situ* longitudinal pre-stretch in the human femoropopliteal artery. Acta Biomater. 32, 231–237. 10.1016/j.actbio.2016.01.002 26766633 PMC4889118

[B41] Khan A. A. Patel J. Desikan S. Chrencik M. Martinez-Delcid J. Caraballo B. (2021). Asymptomatic carotid artery stenosis is associated with cerebral hypoperfusion. J. Vasc. Surg. 73 (5), 1611–1621.e2. 10.1016/j.jvs.2020.10.063 33166609 PMC8209736

[B42] Kleipool E. E. F. Trappenburg M. C. Rhodius-Meester H. F. M. Lemstra A. W. van der Flier W. M. Peters M. J. L. (2019). Orthostatic hypotension: an important risk factor for clinical progression to mild cognitive impairment or dementia. the amsterdam dementia cohort. J. Alzheimers Dis. 71 (1), 317–325. 10.3233/jad-190402 31381517 PMC6839486

[B43] Labrosse M. R. Beller C. J. Mesana T. Veinot J. P. (2009). Mechanical behavior of human aortas: experiments, material constants and 3-D finite element modeling including residual stress. J. Biomech. 42 (8), 996–1004. 10.1016/j.jbiomech.2009.02.009 19345356

[B44] Labrosse M. R. Gerson E. R. Veinot J. P. Beller C. J. (2013). Mechanical characterization of human aortas from pressurization testing and a paradigm shift for circumferential residual stress. J. Mech. Behav. Biomed. Mater 17, 44–55. 10.1016/j.jmbbm.2012.08.004 23127625

[B45] Lelovas P. P. Kostomitsopoulos N. G. Xanthos T. T. (2014). A comparative anatomic and physiologic overview of the porcine heart. J. Am. Assoc. Lab. Anim. Sci. 53 (5), 432–438. 25255064 PMC4181683

[B46] Lipton P. (1999). Ischemic cell death in brain neurons. Physiol. Rev. 79 (4), 1431–1568. 10.1152/physrev.1999.79.4.1431 10508238

[B47] Liu H. Zhang J. (2012). Cerebral hypoperfusion and cognitive impairment: the pathogenic role of vascular oxidative stress. Int. J. Neurosci. 122 (9), 494–499. 10.3109/00207454.2012.686543 22519891

[B48] Manning K. B. (2012). Biofluid mechanics: the human circulation. New York: Oxford University Press.

[B49] Maule S. Milazzo V. Maule M. M. Di Stefano C. Milan A. Veglio F. (2012). Mortality and prognosis in patients with neurogenic orthostatic hypotension. Funct. Neurol. 27 (2), 101–106. 23158582 PMC3812778

[B50] Mensel B. Heßelbarth L. Wenzel M. Kühn J. Lorbeer R. Völzke H. (2016). Thoracic and abdominal aortic diameters in a general population: MRI-based reference values and association with age and cardiovascular risk factors. Eur. Radiol. 26 (4), 969–978. 10.1007/s00330-015-3926-6 26208859

[B51] Mensel B. Quadrat A. Schneider T. Kuhn J. P. Dorr M. Volzke H. (2014). MRI-based determination of reference values of thoracic aortic wall thickness in a general population. Eur. Radiol. 24 (9), 2038–2044. 10.1007/s00330-014-3188-8 24816934

[B52] Michalowsky B. Kaczynski A. Hoffmann W. (2019). The economic and social burden of dementia diseases in Germany a meta analysis. Bundesgesundheitsbl 62 (8), 981–992. 10.1007/s00103-019-02985-z 31297549

[B53] Mikael L. R. Paiva A. M. G. Gomes M. M. Sousa A. L. L. Jardim P. Vitorino P. V. O. (2017). Vascular aging and arterial stiffness. Arq. Bras. Cardiol. 109 (3), 253–258. 10.5935/abc.20170091 28678931 PMC5586233

[B54] Milazzo V. Di Stefano C. Milan A. Ravera A. Sobrero G. Sabia L. (2015). Cardiovascular complications in patients with autonomic failure. Clin. Auton. Res. 25 (3), 133–140. 10.1007/s10286-015-0275-0 25791260

[B55] Min M. Shi T. Sun C. Liang M. Zhang Y. Wu Y. (2018). The association between orthostatic hypotension and dementia: a meta-analysis of prospective cohort studies. Int. J. Geriatr. Psychiatry 33 (12), 1541–1547. 10.1002/gps.4964 30247788

[B56] Nie J. Hou L. Tan B. (2022). Correlation between carotid stenosis degree and blood pressure variability in patients with carotid stenosis. Comput. Math. Methods Med. 2022, 4305015. 10.1155/2022/4305015 35637843 PMC9148253

[B57] Numata S. Itatani K. Kanda K. Doi K. Yamazaki S. Morimoto K. (2016). Blood flow analysis of the aortic arch using computational fluid dynamics. Eur. J. Cardiothorac. Surg. 49 (6), 1578–1585. 10.1093/ejcts/ezv459 26792932

[B58] Peña J. A. Martínez M. A. Peña E. (2019). Failure damage mechanical properties of thoracic and abdominal porcine aorta layers and related constitutive modeling: phenomenological and microstructural approach. Biomech. Model Mechanobiol. 18 (6), 1709–1730. 10.1007/s10237-019-01170-0 31123879

[B59] Qiu C. von Strauss E. Fastbom J. Winblad B. Fratiglioni L. (2003). Low blood pressure and risk of dementia in the Kungsholmen project: a 6-year follow-up study. Arch. Neurol. 60 (2), 223–228. 10.1001/archneur.60.2.223 12580707

[B60] Rawlings A. Juraschek S. Heiss G. Hughes T. Meyer M. Selvin E. (2017). Abstract 28: orthostatic hypotension is associated with 20-year cognitive decline and incident dementia: the atherosclerosis risk in communities (ARIC) study. Circulation 135 (Suppl. l_1), A28. 10.1161/circ.135.suppl_1.28

[B61] Robertson A. D. Udow S. J. Espay A. J. Merola A. Camicioli R. Lang A. E. (2019). Orthostatic hypotension and dementia incidence: links and implications. Neuropsychiatr. Dis. Treat. 15, 2181–2194. 10.2147/ndt.S182123 31447560 PMC6683958

[B62] Rouch L. Vidal J. S. Hoang T. Cestac P. Hanon O. Yaffe K. (2020). Systolic blood pressure postural changes variability is associated with greater dementia risk. Neurology 95 (14), e1932–e1940. 10.1212/wnl.0000000000010420 32690802 PMC7682838

[B63] Rutan G. H. Hermanson B. Bild D. E. Kittner S. J. LaBaw F. Tell G. S. (1992). Orthostatic hypotension in older adults. The cardiovascular health study. CHS collaborative research group. Hypertension 19 (6 Pt 1), 508–519. 10.1161/01.hyp.19.6.508 1592445

[B64] Saz-Lara A. Cavero-Redondo I. Martínez-Vizcaíno V. Lucerón-Lucas-Torres M. Pascual-Morena C. Sequí-Domínguez I. (2023). Association between arterial stiffness and orthostatic hypotension: a systematic review and meta-analysis. Front. Physiol. 14, 1164519. 10.3389/fphys.2023.1164519 37250126 PMC10210150

[B65] Siebler M. Sitzer M. Rose G. Steinmetz H. (1996). Microembolism in carotid artery disease. Echocardiography 13 (5), 529–536. 10.1111/j.1540-8175.1996.tb00931.x 11442965

[B66] Siesjö B. K. (1992). Pathophysiology and treatment of focal cerebral ischemia. Part I: pathophysiology. J. Neurosurg. 77 (2), 169–184. 10.3171/jns.1992.77.2.0169 1625004

[B67] Singh V. Cheng R. (2021). Neurovascular physiology and neurocritical care. Handb. Clin. Neurol. 176, 71–80. 10.1016/b978-0-444-64034-5.00014-6 33272411

[B68] Skoog I. Lernfelt B. Landahl S. Palmertz B. Andreasson L. A. Nilsson L. (1996). 15-year longitudinal study of blood pressure and dementia. Lancet 347 (9009), 1141–1145. 10.1016/s0140-6736(96)90608-x 8609748

[B69] Sonnesyn H. Nilsen D. W. Rongve A. Nore S. Ballard C. Tysnes O. B. (2009). High prevalence of orthostatic hypotension in mild dementia. Dement. Geriatr. Cogn. Disord. 28 (4), 307–313. 10.1159/000247586 19828952

[B70] Soysal P. Veronese N. Smith L. Torbahn G. Jackson S. E. Yang L. (2019). Orthostatic hypotension and health outcomes: an umbrella review of observational studies. Eur. Geriatr. Med. 10 (6), 863–870. 10.1007/s41999-019-00239-4 34652770

[B71] Tarumi T. Khan M. A. Liu J. Tseng B. M. Parker R. Riley J. (2014). Cerebral hemodynamics in normal aging central artery stiffness, wave reflection, and pressure pulsatility. J. Cereb. Blood Flow Metabolism 34, 971–978. 10.1038/jcbfm.2014.44 PMC4050241 24643081

[B72] Thomas B. Sumam K. S. (2016). Blood flow in human arterial system-a review. Procedia Technol. 24, 339–346. 10.1016/j.protcy.2016.05.045

[B73] Tilvis R. S. Hakala S. M. Valvanne J. Erkinjuntti T. (1996). Postural hypotension and dizziness in a general aged population: a four-year follow-up of the Helsinki Aging Study. J. Am. Geriatr. Soc. 44 (7), 809–814. 10.1111/j.1532-5415.1996.tb03738.x 8675929

[B74] Töyry J. P. Kuikka J. T. Länsimies E. A. (1997). Regional cerebral perfusion in cardiovascular reflex syncope. Eur. J. Nucl. Med. 24 (2), 215–218. 10.1007/bf02439557 9021122

[B75] Vloet L. C. Pel-Little R. E. Jansen P. A. Jansen R. W. (2005). High prevalence of postprandial and orthostatic hypotension among geriatric patients admitted to Dutch hospitals. J. Gerontol. A Biol. Sci. Med. Sci. 60 (10), 1271–1277. 10.1093/gerona/60.10.1271 16282558

[B76] WHO (2021). Global status report on the public health response to dementia: executive summary. Geneva: World Health Organization.

[B77] Wilkins K. Campbell N. R. Joffres M. R. McAlister F. A. Nichol M. Quach S. (2010). Blood pressure in canadian adults. Health Rep. 21 (1), 37–46. 20426225

[B78] Xia X. Qiu C. Rizzuto D. Fratiglioni L. Dai L. Laukka E. J. (2023). Role of orthostatic hypotension in the development of dementia in people with and without cardiovascular disease. Hypertension 80 (7), 1474–1483. 10.1161/hypertensionaha.123.21210 37203439 PMC10262990

[B79] Xing C. Y. Tarumi T. Liu J. Zhang Y. Turner M. Riley J. (2017). Distribution of cardiac output to the brain across the adult lifespan. J. Cereb. Blood Flow. Metab. 37 (8), 2848–2856. 10.1177/0271678x16676826 27789785 PMC5536794

[B80] Yap P. L. Niti M. Yap K. B. Ng T. P. (2008). Orthostatic hypotension, hypotension and cognitive status: early comorbid markers of primary dementia? Dement. Geriatr. Cogn. Disord. 26 (3), 239–246. 10.1159/000160955 18841007

