# Supplementary material for: Quantitative analysis of systemic perfusion and cerebral blood flow in the modeling of aging and orthostatic hypotension
Source: Front Physiol. 2024 Aug 1;15:1353768. doi: 10.3389/fphys.2024.1353768 (PMC11324494; doi:10.3389/fphys.2024.1353768)
Supplement: Supplementary file 1 [file DataSheet1.docx]

Supplementary Material

# Supplementary Figures and Tables

## Supplementary Figures


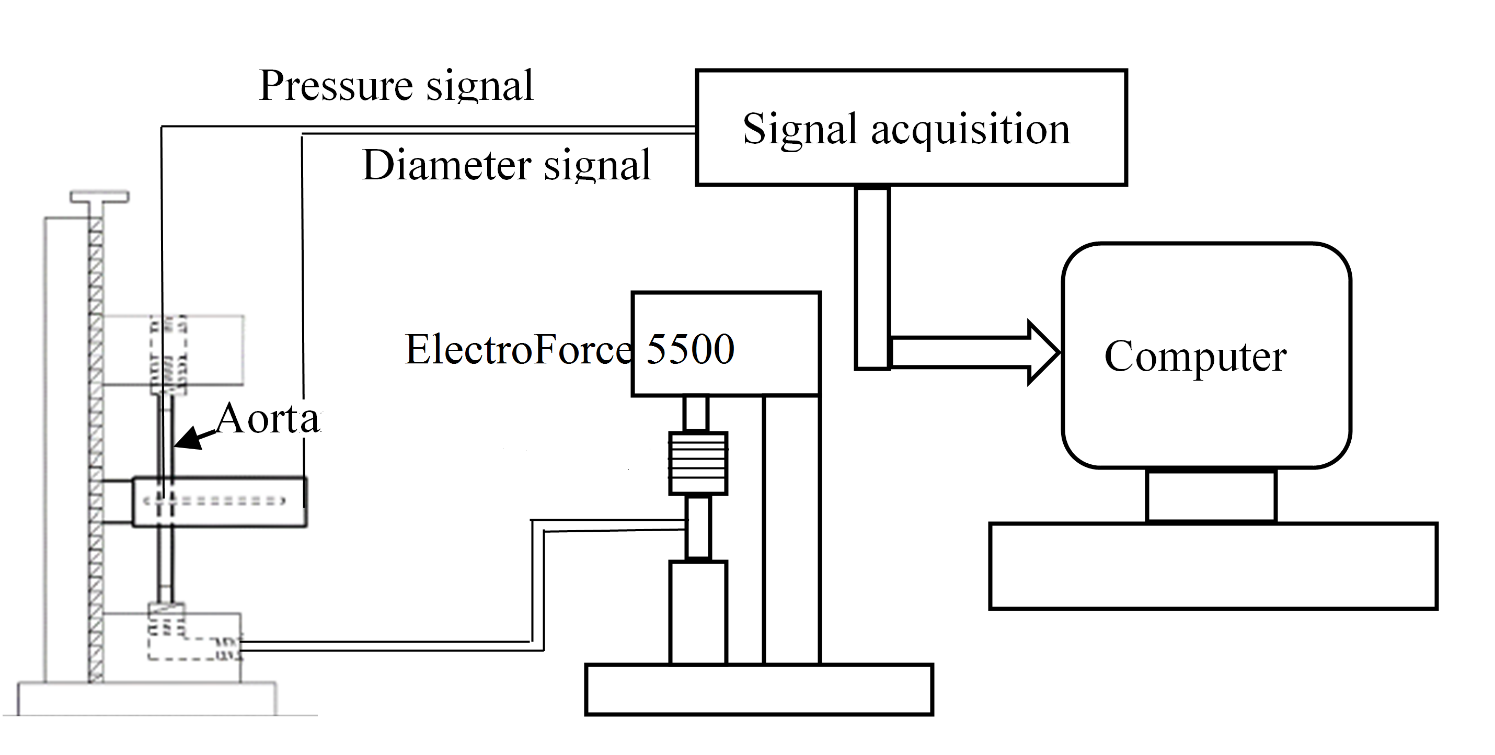


**Fig. S1** Arterial vessel aging simulation device.

## Supplementary Tables

**Table S1 Geometric dimensions of human and porcine thoracic aortas at different AAPSRs**

| Age  (yr) | AAPSR ^1^  *λz* | outer diameter  (mm) | | inner diameter  (mm) | | wall thickness  (mm) | |
| --- | --- | --- | --- | --- | --- | --- | --- |
| Human | Human/Porcine | Human | Porcine | Human | Porcine | Human | Porcine |
| 20 | *λ_z_*=1.33 | 16.91 | 17.04±1.62 | 14.07 | 12.79±1.23 | 1.42 | 2.12±0.38 |
| 30 | *λ_z_*=1.23 | 18.10 | 17.76±1.67 | 15.08 | 13.36±1.36 | 1.51 | 2.20±0.40 |
| 60 | *λ_z_*=1.08 | 21.63 | 19.72±1.80 | 18.11 | 15.27±1.46 | 1.76 | 2.22±0.43 |
| 70 | *λ_z_*=1.05 | 22.79 | 20.03±1.37 | 19.13 | 15.52±1.87 | 1.83 | 2.25±0.59 |
| 80 | *λ_z_*=1.01 | 23.08 | 20.62±1.41 | 19.40 | 16.03±1.68 | 1.84 | 2.29±0.63 |

Note: AAPSR means arterial axial pre-stretch rate.

1. Horny, L., Adamek, T., and Kulvajtova, M. Analysis of axial prestretch in the abdominal aorta with reference to post mortem interval and degree of atherosclerosis. *J Mech Behav Biomed Mater.* 2014, 33: 93-98. doi: 10.1016/j.jmbbm.2013.01.033
